# A Causal Regulation Modeling Algorithm for Temporal Events with Application to *Escherichia coli*’s Aerobic to Anaerobic Transition

**DOI:** 10.3390/ijms25115654

**Published:** 2024-05-22

**Authors:** Yigang Chen, Runbo Mao, Jiatong Xu, Yixian Huang, Jingyi Xu, Shidong Cui, Zihao Zhu, Xiang Ji, Shenghan Huang, Yanzhe Huang, Hsi-Yuan Huang, Shih-Chung Yen, Yang-Chi-Duang Lin, Hsien-Da Huang

**Affiliations:** 1 School of Medicine, The Chinese University of Hong Kong, Shenzhen, Longgang District, Shenzhen 518172, China; yigangchen@link.cuhk.edu.cn (Y.C.); runbomao@link.cuhk.edu.cn (R.M.); jiatongxu@link.cuhk.edu.cn (J.X.); yixianhuang@link.cuhk.edu.cn (Y.H.); jingyixu@link.cuhk.edu.cn (J.X.); shidongcui@link.cuhk.edu.cn (S.C.); zihaozhu1@link.cuhk.edu.cn (Z.Z.); xiangji@link.cuhk.edu.cn (X.J.); shenghanhuang@link.cuhk.edu.cn (S.H.); yanzhehuang@link.cuhk.edu.cn (Y.H.); huanghsiyuan@cuhk.edu.cn (H.-Y.H.); jonesyen@cuhk.edu.cn (S.-C.Y.); 2 Warshel Institute for Computational Biology, School of Medicine, The Chinese University of Hong Kong, Shenzhen, Longgang District, Shenzhen 518172, China

**Keywords:** causal signaling model, temporal transcriptomics, *Escherichia coli*, aerobic-to-anaerobic transition, biological modeling

## Abstract

Time-series experiments are crucial for understanding the transient and dynamic nature of biological phenomena. These experiments, leveraging advanced classification and clustering algorithms, allow for a deep dive into the cellular processes. However, while these approaches effectively identify patterns and trends within data, they often need to improve in elucidating the causal mechanisms behind these changes. Building on this foundation, our study introduces a novel algorithm for temporal causal signaling modeling, integrating established knowledge networks with sequential gene expression data to elucidate signal transduction pathways over time. Focusing on *Escherichia coli’*s (*E. coli*) aerobic to anaerobic transition (AAT), this research marks a significant leap in understanding the organism’s metabolic shifts. By applying our algorithm to a comprehensive *E. coli* regulatory network and a time-series microarray dataset, we constructed the cross-time point core signaling and regulatory processes of *E. coli*’s AAT. Through gene expression analysis, we validated the primary regulatory interactions governing this process. We identified a novel regulatory scheme wherein environmentally responsive genes, *soxR* and *oxyR*, activate *fur*, modulating the nitrogen metabolism regulators fnr and nac. This regulatory cascade controls the stress regulators *ompR* and *lrhA*, ultimately affecting the cell motility gene *flhD*, unveiling a novel regulatory axis that elucidates the complex regulatory dynamics during the AAT process. Our approach, merging empirical data with prior knowledge, represents a significant advance in modeling cellular signaling processes, offering a deeper understanding of microbial physiology and its applications in biotechnology.

## 1. Introduction

Biological processes are transient and dynamic, and time-series experiments are essential tools for investigating cellular processes such as cell cycle progression [1,2] and immune responses [3]. Monitoring transcriptomics data at various time points allows researchers to uncover insights into the systematic responses, enhanced by the progression in data analysis techniques. Clustering algorithms, including hierarchical clustering [4], k-means [5], and self-organizing maps [6], play a crucial role in this analysis—these methods group genes with similar expression patterns, elucidating gene functions and their interactions.

Based on the clustering algorithms, time-series data enable the inference of causality through causal modeling [7]. This approach analyzes sequences of gene expression to infer relationships between genes. The initial methods focused on aligning expression patterns to uncover either similar or opposite sequences, helping to identify potential gene activators and repressors [8]. Subsequently, more sophisticated techniques, such as regression analysis, have advanced the understanding of gene interactions. This is evidenced by studies on zebrafish somitogenesis, highlighting the sequential activation of transcription factors [9,10].

Current methods for analyzing time-series data primarily focus on constructing gene regulatory networks (GRNs) based on observed gene expression data from experiments [11]. However, these approaches often overlook a critical aspect of biological regulation: the upstream regulatory processes that lead to the observed gene expressions are fundamental to understanding biological complexities. There is a pressing need for refined temporal analysis models that not only capture the observed expression patterns but also unravel the underlying regulatory mechanisms preceding these patterns. Such models would offer a more comprehensive view of cellular behavior and gene interactions over time, significantly advancing our understanding of biological processes.

In the field of causal modeling, particularly within molecular biology, recent developments have introduced computational techniques that integrate omics data with prior knowledge to elucidate signaling network states in specific contexts [12]. These methods integrate conventional causal inference frameworks with prior knowledge networks (PKNs), which may be signed or unsigned, to construct or rank cellular signaling networks. This approach aims to interpret observed omics patterns mechanistically. Key algorithms employed in modeling signal transduction include edge filtering and shortest path algorithms such as CausalR [13] and NicheNet [14], pinpointing direct signaling routes. Graph theory and statistical testing methods such as CLIPPER [15] and sub-SPIA [16] analyze network structures and pathway significances. Additionally, integer linear programming (ILP) techniques like TPS [17] and CARNIVAL [18] refine the representation of signaling networks to fit observed data. Collectively, these methodologies enhance our understanding of cellular signaling processes by integrating empirical data with established biological knowledge, offering insights into the complex mechanisms of signal transduction.

*Escherichia coli* (*E. coli*) has long been a cornerstone in biological research due to its ubiquity, genetic tractability, and well-characterized physiology [19]. As a Gram-negative bacterium residing in the intestines of warm-blooded organisms, *E. coli* has been pivotal in elucidating fundamental cellular processes [20]. Its metabolic behavior, particularly in nutrient utilization and energy production, has been a focal point for researchers seeking to unravel the intricacies of microbial physiology. Moreover, *E. coli*’s metabolic versatility in both aerobic and anaerobic conditions makes it an invaluable model for biotechnological applications, such as the production of biofuels and pharmaceuticals [21]. Thus, delving into the metabolic intricacies of *E. coli* is imperative for advancing our understanding of microbial biology and harnessing its potential for various scientific and industrial endeavors.

Due to its facultative nature, *E. coli* displays distinct metabolic behaviors under aerobic and anaerobic conditions. During aerobic conditions, *E. coli* primarily relies on glucose metabolism, which flows through glycolysis and directs towards the TCA cycle for higher ATP generation [22]. In anaerobic settings, pyruvate formatelyase or lactate dehydrogenase alters pyruvate into formate, acetyl-CoA, lactate, succinate, acetate, or ethanol. These pathways collectively limit metabolic fluxes to the TCA cycle [21]. Consequently, significant metabolic transitions transpire during aerobiosis or the aerobic to anaerobic transition (AAT), which involves complex regulatory mechanisms that orchestrate metabolic shifts to adapt to changing environmental conditions.

The AAT in *E. coli* is characterized by dynamic adjustments in gene expression, enzyme activity, and metabolic fluxes [23]. Previous research has employed various experimental approaches to decipher the regulatory events contributing to *E. coli*’s metabolic adaptation [24,25]. Key regulators such as the ArcA/B and FNR systems were identified to play pivotal roles in coordinating the transition by sensing and responding to changes in oxygen availability [26]. Recent research on AAT combined transcriptomic data and a multi-dimensional statistic model to decipher the interplay of genes during the AAT transition [27]. Cameron and colleagues developed a machine learning module specifically for analyzing RNA-seq data related to the AAT, highlighting the system-level changes during the anaerobic to aerobic transition [25]. These studies have provided insights into the dynamic changes during AAT but still have limitations, including lacking exhaustive expression profiles, limiting regulatory network knowledge, and time-consuming computational processes. Therefore, to illustrate the inherent complexity of cellular regulatory networks and the interplay of multiple factors during the AAT, we integrated time-series experiments and temporal causal signaling modeling methods to resolve transient and subtle changes in cellular physiology.

In this study, we present a novel algorithm for temporal causal signaling modeling. This algorithm is specifically designed to infer signal transduction pathways by merging prior knowledge networks with time-series gene expression data. To demonstrate our algorithm, we developed a signal transduction prior knowledge network of *E. coli* and generated a time-series gene expression dataset capturing the organism’s transition from aerobic to anaerobic environments. By applying our temporal causal signaling model, we identified crucial regulatory elements and elucidated the mechanisms underlying *E. coli*’s AAT.

## 2. Results

### 2.1. Overview of This Study

**Figure 1:**
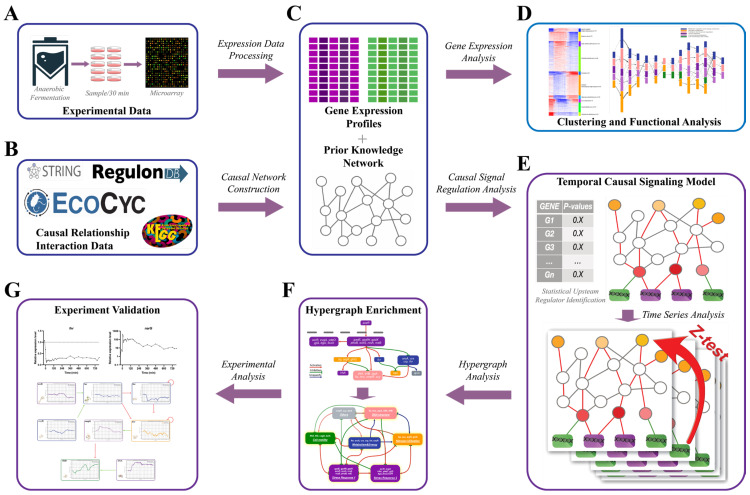
The workflow of this research. (**A**) Experimental data collection: Time-series microarray experiments were performed to study *E. coli*’s aerobic to anaerobic transition (AAT), sampling every 30 min. (**B**) Prior knowledge collection: Causal relationship interaction data were collected from various databases. (**C**) Data integration: Time-series gene expression data of *E. coli*’s AAT were processed, and a comprehensive prior knowledge network of *E. coli* was constructed. (**D**) Functional analysis: Functional analysis using k-means and GO information flow network was conducted to describe the overall process of *E. coli*’s AAT. (**E**) Temporal causal signaling model: The combined gene expression data and prior knowledge network were utilized to model the signal transduction processes in *E. coli*’s AAT through a temporal causal signaling model. (**F**) Hypergraph enrichment: A hypernode enrichment method was employed to group nodes for enhanced visualization and analysis. (**G**) Experimental validation: The regulatory hypotheses inferred from the model were validated using results from gene expression experiments.

In this study, we introduce a novel algorithm designed to analyze the dynamics of signal transduction pathways by integrating established knowledge networks with sequential gene expression data. We demonstrated the capabilities of our temporal causal signaling model by using it to construct a detailed regulatory network for *E. coli*’s AAT. This model enabled us to identify key regulatory elements and elucidate the mechanisms governing the AAT (Figure 1).

Initially, we gathered experimental data through time-series microarray experiments that tracked *E. coli* gene expression at 30 min intervals during the AAT. After processing these data to cluster and identify differentially expressed genes (DEGs), we built a gene ontology information flow network to explore the functional dynamics during this transition. Concurrently, we compiled a comprehensive causal network of *E. coli* regulation from various sources, which served as prior knowledge.

We then applied the temporal causal signaling model to the gene expression profiles and the causal network, facilitating the statistical identification of upstream signal transduction processes over time. This involved tracing the pathways from primary regulatory drivers through signal transduction cascades to their effects on transcription factors (TFs) across different time periods. Additionally, the model considered cross-time callback events to pinpoint the most significant signal transductions throughout the entire process. Thus, we were able to construct a comprehensive network of the most crucial signal transduction pathways involved in the entire *E. coli*’s AAT.

To enhance our analysis, we employed hypergraph enrichment techniques to highlight critical regulatory elements. To confirm the reliability of our microarray data and the regulatory mechanisms proposed, we corroborated the microarray findings with PCR results, ensuring data reproducibility. Changes in gene expression were also used to validate the core regulatory mechanisms suggested by our algorithm.

This approach not only verified the robustness of our model but also provided in-depth insights into the dynamic regulatory events that govern *E. coli*’s transition from aerobic to anaerobic states.

### 2.2. The Basic Temporal DEG- and Clustering-Based Functional Analysis of E. coli’s AAT Process

**Figure 2:**
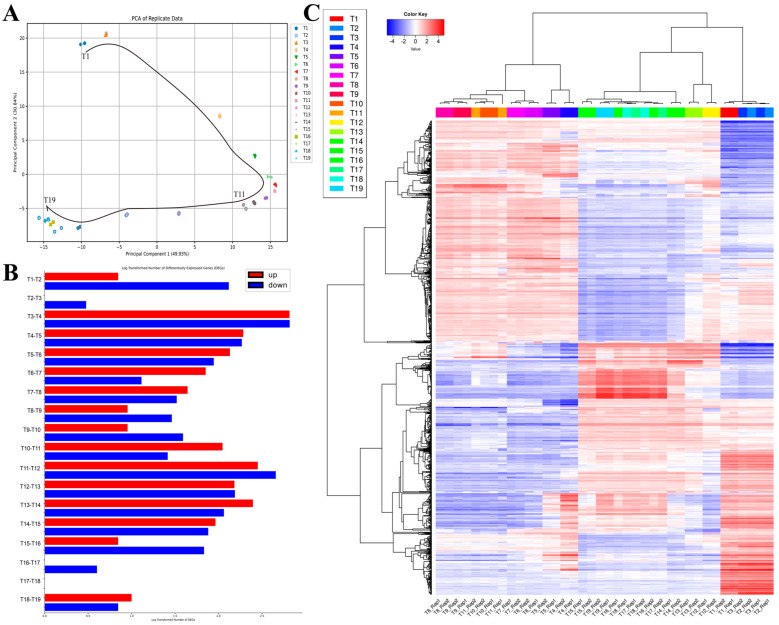
Gene expression profile analysis result. (**A**) PCA results illustrating that all samples demonstrate a smooth overall temporal change. The direction of change is indicated by an arrow. (**B**) The number of differentially expressed genes (DEGs) in log transformation between all adjacent time-point groups. (**C**) Hierarchical clustering analysis results demonstrating the AAT process, which can be distinctly clustered into three periods: T1–T3, T4–T11, and T12–T19.

In our time-series microarray analysis of *E. coli*’s AAT, we have taken a structured approach to examine the changes in gene expression. Initially, we normalized the data, ensuring that the mean expression value was aligned at 10. The subsequent principal component analysis (PCA) revealed a high-quality dataset with discernible temporal shifts, validating our experimental and data processing methods (Figure 2A).

Differential expression analysis between adjacent time points highlighted two periods of intense activity: between T3–T4 and T12–T13. This finding suggests critical shifts in gene expression during these intervals (Figure 2B). Hierarchical clustering analysis further supported this, segregating the data into three distinct time clusters. The first period includes T1–T3, the second period includes T4–T11, and third period includes T12–T19. However, the specific functions within each period were not discernible through this method alone (Figure 2C).

**Figure 3:**
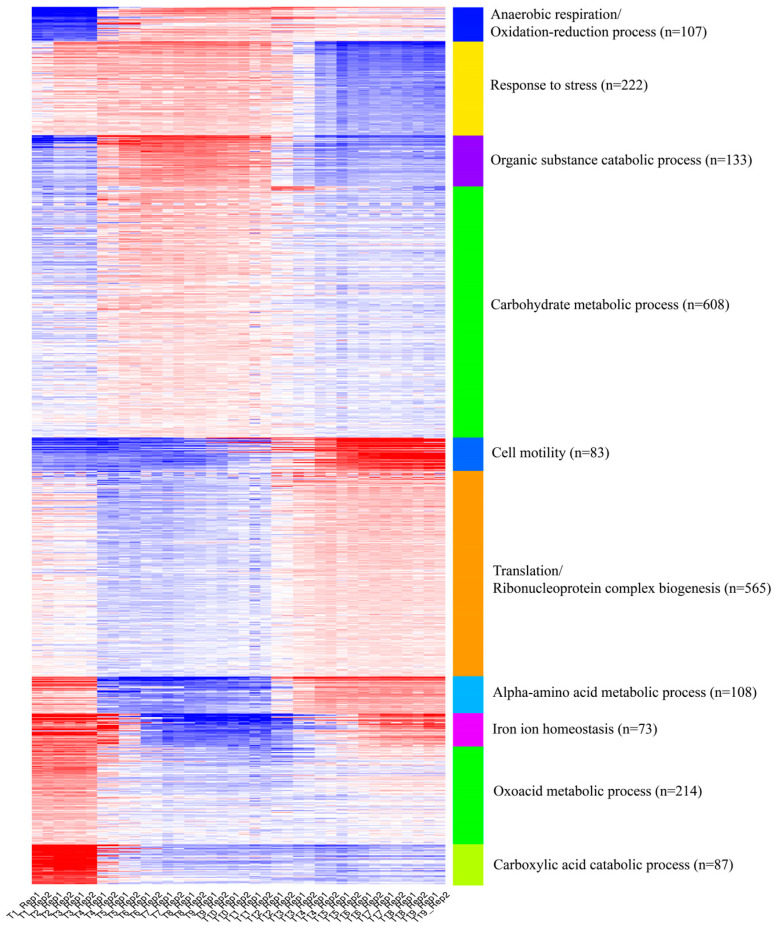
K-means clustering of AAT process. Top 2200 variant genes were collected to generate 10 clusters. Each cluster was annotated by the most significant GO terms.

K-means clustering analysis is commonly used to analyze time-series data. To enhance our understanding of the functional dynamics throughout the timeline, we combined k-means clustering with gene ontology (GO) analysis. This integration helped us uncover significant biological functions of gene groups that exhibit similar expression patterns, thereby providing a deeper interpretation of our data as shown in Figure 3. Initially, our findings suggest that *E. coli* was adjusting to reduced oxygen levels. In the following phase, the bacterium underwent a pronounced anaerobic transition, marked by significant changes in energy metabolism. In the final phase, gene functions stabilized, and the cellular stress response became more regulated, which possibly aided in regulatory adaptations or a return to homeostasis. Concurrently, *E. coli* started modifying its morphology, enhancing its motility, initiating translation and ribonucleoprotein complex biogenesis, and adjusting its iron homeostasis and oxoacid metabolic pathways. Although k-means clustering provides a preliminary overview of the adaptive anaerobic transition (AAT) processes occurring in *E. coli*, it is limited in its ability to delineate the temporal causality of these changes. In the subsequent sections, we will demonstrate how our temporal causal signaling model can articulate a more comprehensive and dynamic representation of the entire process, thereby expanding on the insights gleaned from the clustering analysis.

### 2.3. The Regulatory Network Model of E. coli

A comprehensive PKN is essential for our temporal causal signaling model. To achieve this, we initially constructed an aggregated PKN, encompassing protein interactions, gene regulation, and metabolic processes. This network is intricately detailed, comprising 12,152 nodes, 19,640 interactions, and 1,089,246 correlations. Each interaction within the network is meticulously documented, including information on the regulatory effects, mechanisms of interaction, bibliographic references, data sources, impact factor (IF), and IF-score annotations (Table 1).

### 2.4. Elucidating the Dynamic Regulatory Network in AAT Process through Computational Modeling

**Figure 4:**
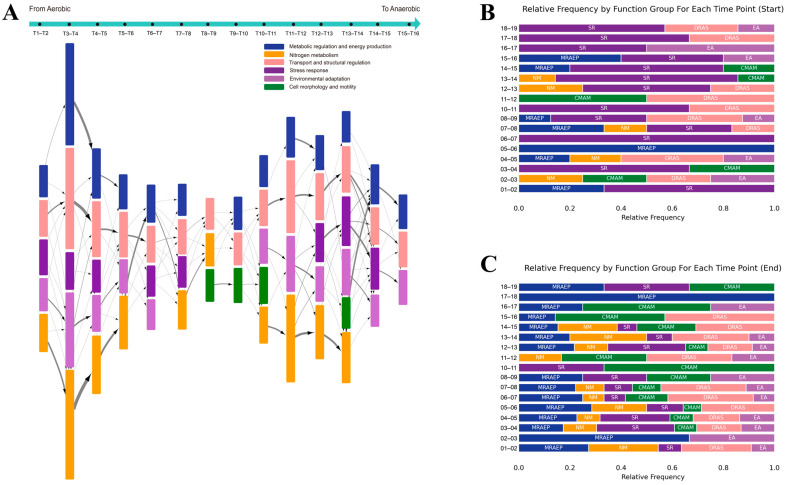
Comparative visualization of the AAT process using functional enrichment and causal inference analyses. (**A**) Gene expression-based GO information flow illustrates the functional transformation of AAT, identifying primary functions of DEGs at each time point. Function-specific colors, block lengths representing the number of DEGs involved, and edge weights reflecting shared DEGs between time points clarify temporal functional changes. (**B**) The temporal causal signaling model outlines the AAT process over 19 time points, focusing on upstream primary regulatory drivers. Each row denotes a time segment with color-coded functions; the extent of each color indicates function frequency. Notably, pink now denotes DNA regulation and structural regulation, diverging slightly from panel A’s color coding. (**C**) Downstream effectors are detailed, highlighting their roles and contributions throughout the AAT process, facilitating a comprehensive understanding of end-stage regulatory effects.

To showcase the capabilities and advantages of our temporal causal signaling model, we initiate a comparative analysis against a modified functional enrichment model for delineating the AAT process. The functional enrichment approach reveals the gene ontology (GO) information flow over time, providing insights into the functional transformations of differentially expressed genes (DEGs) in *E. coli* under oxygen deprivation (Figure 4A). The approach categorizes functions into six main groups. Particularly, nitrogen metabolism was emphasized separately due to its particular importance in the *E. coli* AAT process, highlighting its role in adapting to anaerobic conditions. The edges’ width represents the connections between GO terms at adjacent time points, reflecting the count of DEGs that are common to consecutive time points. Regarding the edges and their thickness, some genes have multiple functions and, therefore, could be represented in more than one GO term category. Such genes form thinner lines connecting different functional groups across time points, indicating their multi-functional role in *E. coli*’s adaptive response. This visualization intuitively captures the functional evolution of *E. coli*’s AAT, highlighting significant functional changes at crucial phases such as T3–T4 and T11–T14. At T3–T4, a broad functional shift occurs, especially in metabolic processes related to organonitrogen compounds, signaling a critical transition to anaerobic metabolism. At T11–T14, changes in response to stimuli suggest a gradual adaptation process.

However, the functional enrichment model lacks the capacity to elucidate the underlying dynamic signal transduction processes that drive direct gene expression changes. To address this, we introduced the temporal causal signaling model. As depicted in overview section, this model applies to the gene expression profiles and the causal network, enabling the statistical identification of upstream signal transduction processes over time. It traces the pathways from primary regulatory drivers through signal transduction cascades to their impacts on transcription factors (TFs) and includes cross-time callback events to highlight significant signal transductions across the entire process, constructing a detailed network of crucial pathways in *E. coli*’s AAT.

The primary regulatory drivers identified typically relate to stress response and DNA structure regulation, with downstream effects prominently involving energy and nitrogen metabolism, and modifications in flagellar morphology and DNA structure regulation (Figure 4B,C). Compared to the functional analysis-based method, the causal signaling model offers several advantages. It provides a more granular explanation of the regulatory processes occurring between time points. For instance, while both methods detect changes in five functions between T1 and T2, the causal model pinpoints the specific causes, such as stress response and metabolic changes. Furthermore, it reveals callback phenomena across time points, where downstream functional changes at an earlier time point become upstream regulatory factors at a later stage. Lastly, the causal model allows for a detailed investigation of key regulatory drivers and their potential temporal regulatory relationships, which will be further discussed in the subsequent sections.

### 2.5. Visualization of the Signal Transduction in AAT Process by Hypernode Enrichment

The temporal causal signaling model delineates all potential signal paths between adjacent time points and utilizes callback phenomena in time-series data to identify key regulatory factors. Despite refining key elements to several dozen nodes, direct structured network visualization remains challenging. To facilitate visualization, we introduced hypernode computations, which cluster certain nodes into a hypernode by analyzing subgraph topology. Thanks to our high-quality PKN, these hypernodes exhibit functional similarity, enhancing visual presentation. Consequently, all signal transduction processes of *E. coli* AAT are constructed using hypernode interactions, as detailed in the Supplementary File.

**Figure 5:**
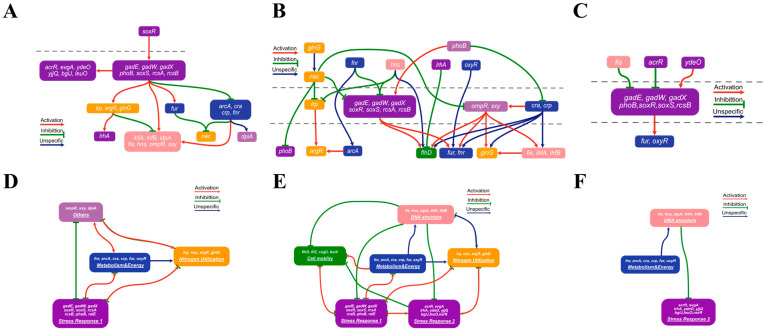
Visualization of signal transduction in the AAT process via hypernode enrichment. This figure displays the causal signal transduction model outcomes at three representative time intervals, enhanced by hypernode enrichment: (**A**) T3–T4, (**B**) T7–T8, and (**C**) T16–T17. Each panel shows the directional flow of signals from upstream to downstream within these intervals. The colors of the nodes distinguish the hypernodes, and the arrows, with colors and shapes that are explained in the legend, denote the types of regulatory relationships. Panels (**D**–**F**) depict integrated signal transduction networks for the three main AAT phases, respectively, the first period (T1–T4), the second period (T4–T12), and the last period (T12–T19), illustrating the comprehensive regulatory networks over the AAT timeline.

During the whole modeling process of AAT, although stress response was not identified by DEG analysis, causal analysis revealed upstream stress regulation leading to downstream shifts in energy metabolism, exemplified that, during time points T3–T4, there exist a significant stress response driven by *soxR*, which influences the downstream energy and nitrogen metabolism regulation (Figure 5A). The primary regulatory processes of the first period are summarized in Figure 5D. 

Intriguingly, during the subsequent phases of sustained stress adaptation and morphological adjustment, the flagellar morphological changes gradually become dominated. For instance, between time points T7–T8, energy and nitrogen metabolism along with stress response were positioned as upstream primary regulatory drivers, culminating in complex downstream regulatory activities, including the regulation of the flagellar control factor *flhD* (Figure 5B), with major regulatory processes summarized in Figure 5E. 

In the final stages, after undergoing morphological transformation, *E. coli* continues to adapt and optimize in response to environmental changes. For instance, the phase T16–T17 is marked by straightforward adaptations to stress and subtle refinements in energy metabolism (Figure 5C). The principal regulatory activities during the final period are concisely summarized in Figure 5F.

This analysis, leveraging causal signaling and temporal path searching methodologies, reveals intricate regulatory dynamics underlying the AAT process, illustrating a sequential response involving initial stress adaptation, followed by energy and nitrogen metabolism adjustments, morphological changes, and culminating in a stabilized adaptation phase.

### 2.6. Validation and Dynamic Analysis of the AAT Regulatory Network through Gene Expression Profiling

**Figure 6:**
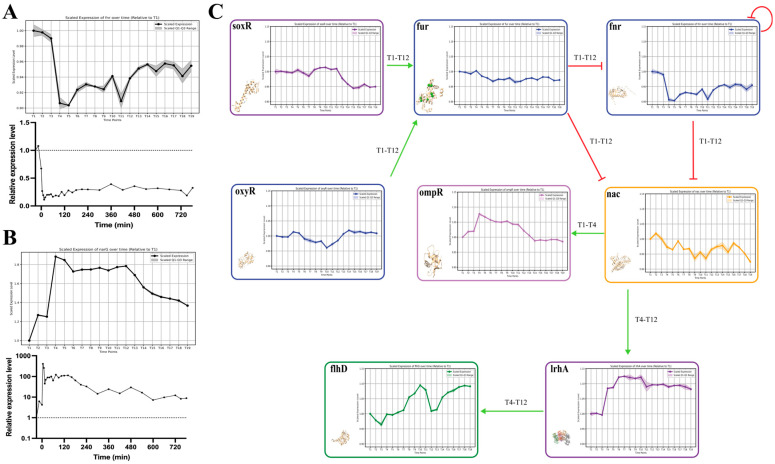
Analysis of gene expression dynamics in the AAT regulation process. This figure demonstrates the consistency between the microarray and qPCR results for key gene expression in the AAT process and corroborates the regulatory outcomes inferenced by the temporal causal signaling model using gene expression data. The graph in (**A**) demonstrates the correlation of expression levels for the marker gene *fnr* as measured by both microarray and qPCR techniques. (**B**) Similar comparative analysis for the downstream regulated gene *narG*. The graphs in (**C**) detail the fluctuations in gene expression of core regulatory genes during the first (T1–T3) and second phases (T4–T12) of the AAT process, validating the complex network of signal transductions proposed by temporal causal signaling model.

Our analysis aimed at validating the reliability of the proposed regulatory network in the AAT process through gene expression analysis. Initially, the accuracy of the microarray experimental results was verified against qPCR data for the AAT marker gene *fnr* and its downstream regulated gene *narG* (Figure 6A,B). The high consistency between microarray and qPCR outcomes affirmed the precision of the microarray data. 

Subsequent in-depth analysis focused on interactions during the most dynamic phases of the AAT process. We identified and illustrated the most frequently occurring significant interactions suggested by our temporal causal signaling model from T1 to T12, showcasing their gene expression patterns, regulatory roles, and the periods during which they were active (Figure 6C).

Specifically, the environmental stress regulatory genes *soxR* and *oxyR* responded promptly to oxygen stress in the AAT process and remained highly expressed throughout. Their combined effect facilitated the continuous high expression of the gene *fur*, which further regulated *fnr* and *nac* from T1 to T12. Notably, the decreasing expression of *fnr* is indicative of the regulatory activation of FNR protein due to its self-regulatory and activating properties. The combined action of *fnr* and *fur* subsequently led to a gradual decline in the expression of *nac* throughout T1 to T12.

Our computational analysis revealed that *nac* primarily activated *ompR* expression during T1–T4 and predominantly triggered *lrhA* expression from T4–T12 (Figure 5D,E). Gene expression data for *ompR* and *lrhA* supported this theory; *ompR* expression was upregulated from T1–T3 and then gradually decreased, while *lrhA* showed minimal changes in the early T1–T3 phase but significantly increased from T4–T12 and maintained high expression levels, validating the interactions suggested by our algorithm.

Finally, during the T4–T12 period, *lrhA*, acting as a key regulatory factor, stimulated the expression of *flhD*, ultimately leading to morphological changes in *E. coli*. This sequence of regulatory events illustrates the sophisticated dynamics of the AAT process and underscores the accuracy and utility of our causal modeling approach.

## 3. Discussion

In this study, we introduced a novel algorithm for temporal causal signal modeling that integrates prior knowledge network (PKN) with temporal transcriptomics data to elucidate the causal regulatory mechanisms underlying biological processes. Utilizing *E. coli*, a model organism known for its straightforward and well-characterized regulatory systems and consistent gene and protein expression profiles, we aimed to shed light on the aerobic to anaerobic transition (AAT) process, which, despite its importance, remains incompletely understood [27]. To achieve this, we constructed an extensive *E. coli*-based PKN and performed temporal transcriptomics experiments to investigate *E. coli*’s AAT. Utilizing our algorithm, we mapped out the regulation mechanism of *E. coli*’s AAT and validated the central regulatory mechanisms identified by our model through the analysis of gene expression patterns.

Contrary to traditional time-series data analysis techniques that largely focus on constructing GRNs [11,28], our methodology integrates PKN and places a greater emphasis on deducing causal upstream regulations across various time points, and especially focusing on modeling the dynamics of signal transduction across these intervals. As indicated by our gene expression analysis results (Figure 2 and Figure 3) and the GO information flow analysis outcomes (Figure 4A), the AAT process in *E. coli* can be distinctly segmented into three phases across T1 to T19. Our temporal causal modeling elaborates on these three periods, offering intricate insights into the entire process. We observed that during the initial phase of *E. coli*’s AAT, the regulatory response to oxidative stress is consistent and surprisingly more specific than in the second phase. We propose that the AAT process transitions from an immediate and specific adaptation to oxidative stress to a more complex adaptation of environmental pressures and bacterial morphological adjustments, culminating in a relatively stable state. Simple analyses of gene expression are inadequate for capturing these dynamic shifts, as methods like k-means clustering might suggest high consistency across phases [29], but our findings dissect the observed data’s underlying causal relationships, presenting a clearer perspective.

The most intriguing aspect of this study lies in our analysis of the key interactions identified in the initial two stages of the AAT process. These interactions, also high-quality connections within the PKN, have been recognized in various biological processes in previous research [30,31,32]. In our study, we further contextualized these relationships within the AAT process, proposing regulatory mechanisms that encompass both the nature of these interactions and their timing, and meticulously validated by observed gene expression patterns. Overall, our algorithm uncovers more nuanced and hidden regulatory relationships, demonstrating its ability to delicately identify the underlying control mechanisms in biological processes.

However, our proposed algorithm also faces several limitations. Firstly, the precision of the information in the PKN and the inherent complexity of biological regulatory networks still pose challenges. For example, our calculations using the breadth-first search (BFS) [33] do not account for the positive or negative nature of regulatory relationships along a path. Taking FNR as an example, its activation is not directly related to its expression levels. Instead, due to its regulatory characteristics, activated FNR can lead to a reduction in the expression levels of the *fnr* gene under certain conditions [34]. A more comprehensive network that includes various cofactors and regulatory details would enable the design of algorithms that consider these regulatory nuances during path calculation. Secondly, due to limitations in the experimental data, constructing quantitative regulatory relationships is challenging. For example, conducting the experiment several times to achieve reliable biological replicates is necessary when determining the likelihood of a specific interaction within a given period or incorporating Bayesian approaches into our path search and modeling effort. Such repeated experiments are crucial for the accurate application of probabilistic statistical analysis.

In summary, this study introduces a novel algorithm for temporal causal signal modeling that seamlessly integrates PKN with temporal transcriptomics data to reveal the causal regulatory mechanisms at the core of biological processes. We successfully decoded the AAT process in *E. coli*, demonstrating the algorithm’s capability to uncover deeper and significant regulatory relationships within time-series data. Moreover, our findings reveal a novel mechanism of *E. coli*’s AAT enhancing our understanding of *E. coli* and potentially aiding in the design of fermentation bioengineering processes. In future research, we aim to construct more sophisticated networks and gather more comprehensive data, refining our algorithm for application in areas such as disease mechanisms, with the hope of discovering more intriguing biological mechanisms.

## 4. Methods and Materials

### 4.1. Microarray and qRT-PCR Gene Expression Experiments

Microarray experiment data were curated from Project No. 102-2815-C-009-025-B, supported by the National Science and Technology Council, Taiwan. We utilized the in-house *E. coli* K12 BW25113 strain, a derivative BD792. This strain was cultivated in a chemostat under meticulously controlled conditions in a modified Vogel-Bonner medium, designed to limit nutrients and thereby induce specific growth rates. We conducted our continuous-culture experiments both aerobically and anaerobically, adjusting the air and nitrogen flow rates to maintain the desired oxygen levels. The accuracy of the environmental conditions and the attainment of steady-state gene expression were verified by β-galactosidase activity assays (Abcam Inc., Cambridge, MA, USA). Samples of *E. coli* undergoing AAT conversion were collected every 30 min for microarray analysis. The data processing for these experiments employed the Robust Multi-chip Average (RMA) algorithm, which yielded log2-transformed expression values. For gene expression analysis, real-time PCR was conducted using specific conditions and controls, with the results being processed and analyzed through LightCycler 480 software (version 1.5.0.39). Comprehensive descriptions of these methods are available in the Supplementary Methods.

### 4.2. E. coli Regulatory Network Model Construction

We developed an intricate model of *E. coli* regulatory mechanisms by merging multi-omics datasets from the EcoCyc [35,36] database for *E. coli* strain K12, enriched by further data from RegulonDB [37] and STRING [38]. This PKN includes a detailed map of genes, their products, chemical entities, and a variety of biological functions, capturing essential processes like gene expression, transcriptional regulation, protein activation, and complex formations. To validate the network’s accuracy, we meticulously assessed the reliability of connections through literature reviews and extracted confidence scores from EcoCyc and RegulonDB. This involved calculating an influence factor (IF) score for each network edge, with those scoring below 0.86 marked to indicate lesser reliability, thereby maintaining the network’s integrity. Recognizing the limitations of traditional network analysis in representing complex biological interactions, our model employs virtual edges to simplify complex nodes into basic units and marks their interactions as “virtual”. This approach not only preserves the network’s biological authenticity but also enhances its analytical tractability.

### 4.3. Gene Expression Analysis Workflow

In this study, we conducted gene expression analysis using the IDEP platform (version 2.1) [39], following a structured approach. Initially, we preprocessed the data to enhance its quality, then performed differential expression analysis with stringent criteria (FDR = 0.1, fold change = 1.5) to identify significantly altered genes. Principal component analysis (PCA) and hierarchical clustering were applied to the top 1000 variable genes to visualize patterns and group genes with similar expression profiles, using correlation distance, average linkage, and a Z-score cutoff of 4 for robust clustering. Additionally, k-means clustering was utilized on 2200 variable genes, dividing them into 10 distinct clusters with mean center normalization to compare expression patterns across clusters. Following the k-means clustering, we conducted gene ontology (GO) biological process enrichment analysis for each of the 10 clusters to identify overrepresented biological processes within each group [40]. This streamlined methodology allowed for an in-depth exploration of gene expression, facilitating the understanding of complex biological processes and functions.

### 4.4. Gene Ontology Information Flow Network

To construct the gene ontology (GO) information flow network, our approach begins by utilizing the QuickGO database to identify all biological process (BP) GO terms and their ancestors linked with each group of differentially expressed genes (DEGs) [40,41]. We then organize these terms and their ancestors based on their coverage to understand the distribution of GO terms across the dataset.

The core of our method involves a greedy algorithm, similar to the approach we detailed in our previous study [42]. Here, we briefly describe its application. This algorithm starts by excluding the top GO terms with the highest coverage to avoid overly general terms. It proceeds by iteratively selecting GO terms that add the most unique UniProt identifiers not already covered by previously chosen terms. This process continues until it selects GO terms that do not meet both conditions of adding a sufficient number of new genes—above a certain threshold—and having limited overlap with already selected genes. Our goal is to balance the coverage of significant genes with the inclusion of a diverse set of genes. The selection ends when adding new GO terms does not significantly increase this diversity. This results in a curated list of GO terms that represents a broad and comprehensive set of genes suitable for further biological analysis. This strategy ensures detailed coverage of the functional aspects of DEGs and aids in identifying specific biological processes relevant to this study.

Finally, we represent each selected GO term at each time point as a node. We measure the connectivity between nodes based on the number of shared genes between adjacent time points and use Cytoscape (version 3.10.0) to visualize the GO information flow network. This methodology emphasizes a clear, logical, and succinct description of the process to map out the functional landscape of DEGs comprehensively.

### 4.5. Causal Signaling Model Algorithm

The algorithm is designed to uncover the causal mechanisms behind observed changes in gene expression over two time periods. It consists of three key components: identifying primary regulatory drivers between the two time points, enriching the significant transcription factors (TFs) within the same timeframe, and constructing the signal transduction pathways that connect these upstream regulators to the TFs.

To elucidate the primary regulatory drivers behind gene expression changes in *E. coli*, our study introduces a novel algorithm that exploits the dysregulation insights offered by the DeMAND approach [43], tailored to our specific gene–gene correlation PKN constructed from STRING database resources. This methodology focuses on uncovering upstream regulators, highlighting the significance of both correlated and long-distance regulatory mechanisms.

Central to our analysis is the application of Kullback–Leibler divergence (KLD) calculations, comparing gene expression profiles between control and perturbed groups to quantify the extent of regulatory dysregulation. This involves aggregating the *p*-values across all interactions associated with each gene to provide a comprehensive measure of dysregulation. Recognizing the necessity for multiple gene expression comparisons, our model generates a synthetic third dataset from the original pair, enabling smoother data distribution and enhancing the robustness of our analysis. Prior to executing KLD computations, we incorporate principal component analysis to circumvent the potential issues related to singular covariance matrices that could arise during Gaussian transformations. This preparatory step ensures the integrity and accuracy of our dysregulation measurements. The procedural intricacies of our algorithm are delineated in Algorithm 1.
**Algorithm 1:** Primary regulatory drivers dysregulation analysis.Inputs: -**Gene Expression Profile:** Ensure at least three samples for both timepoints -**Gene Regulatory Network**
Procedure: -**Average Expression Calculation:** Generate the third group of gene expression values at each timepoint -**Adjacent Timepoints Selection:** Perform gene dysregulation analysis on every two adjacent timepoints in each iteration -**Probability Distribution Estimation:** Replace each expression with a 2-dimensional Gaussian point and estimate the distribution for timepoint 1 and timepoint 2 samples by summing up the gaussians in each group. -**Edge Dysregulation Evaluation:** For two genes *i*, *j* that connect an edge in the, calculate the KLD of the edge by
(1)$$KLD\left(P_{ij}^{1},P_{ij}^{2}\right)=\frac{KLD\left(P_{ij}^{1}|P_{ij}^{2}\right)+KLD\left(P_{ij}^{2}|P_{ij}^{1}\right)}{2},$$
where: (2)$$KLD\left(P_{ij}^{1}|P_{ij}^{2}\right)=\sum_{k=1}^{M}\sum_{l=1}^{M}P_{ij}^{1}\left(k,l\right)\log\left(\frac{P_{ij}^{1}\left(k,l\right)}{P_{ij}^{2}\left(k,l\right)}\right)\;\;\;\;\;\;\;\;\;\;\;\;\;\;\;\;\;\;\;$$
M=Total number of samples$\left(k,l\right)=Sample\;pairs\;between\;two\;timepoints$ -**Hypothesis Testing on Edge Dysregulation:** Create a null model by choosing two genes at random and calculating their KLD repeatedly for 10,000 times, assuming these genes do not share a common edge. *p*-value of each KLD is determine by: (3)$$P_{v_{ij}}=\frac{The\;number\;of\;times\;KLD_{null}\geq KLD\left(P_{ij}^{1},P_{ij}^{2}\right)}{The\;total\;number\;of\;values\;in\;the\;null}\;$$
 -**Gene Dysregulation Determination:** Derive the *p*-value of gene dysregulation by integrating the *p*-values of all the interactions the gene involved using Fisher’s method, (4)$\chi^{2}=\sum_{G_{i↔}G_{j}\in \varepsilon }-2logP_{v_{ij}},\;$
and Brown’s method for correction
Output: Timepoint-dependent dysregulating gene lists, along with their associated *p*-values, revealing insights into the temporal gene regulatory patterns and overarching regulators.


We further developed an enhanced Fisher’s exact test (FET)-based regulatory impact analysis to identify key transcription factors (TFs) influencing gene expression changes. This approach integrates essential components of the URA framework [44], especially focusing on direct transcriptional interactions. Our method examines how a gene’s differential expression correlates with a regulator’s effect, using a modified version of the Fisher’s exact test designed for our specific analysis needs. It calculates *p*-scores to indicate the likelihood of regulatory interactions, where lower *p*-scores suggest stronger links between differentially expressed genes (DEGs) and the regulator’s targets.

By considering the activation or repression states of upstream regulators, our algorithm captures the complexity of gene regulation. It pinpoints influential TF regulators and assesses their impact on downstream gene expression based on a randomized gene set as the null hypothesis in our statistical testing. The detailed procedures are shown in Algorithm 2.
**Algorithm 2:** Enhanced Fisher’s exact test (FET)-based regulatory impact analysis.Inputs: -**Differentially Expressed Genes (DEGs):** Partitioned into two subsets, up-regulated and down-regulated. -**Gene Regulatory Network (PKN)**
Procedure: -**DEG Filtering:** Isolate DEGs that are present within the PKN and maintain their categorization as up or down-regulated. -**Regulatory Node Identification:** Associate DEGs with their immediate upstream regulatory nodes within the PKN. -**Pseudo Activation State Assignment:** Assign a pseudo activation state to each regulator, where each regulator is evaluated twice—once as if it were activated and once as if it were repressed. -**Parameter Calculation:** For a given regulatory node r under a specific pseudo activation state, calculate the ABCD parameters where:n=Total Number of Nodes in PKNa=Number of nodes regulated by r∩DEGs match the predicted regulatory patternb=DEGs match the predicted regulatory pattern ∩Nodes in PKN−ac=Number of nodes regulated by r−ad=n−a−b−c -**Statistical Significance Assessment via Modified FET:**Conduct a modified Fisher Exact Test (FET) using the parameters for each regulators to determine the statistical significance of its regulatory influence. The assessment involves computing the *p*-value p(r) as follows: (5)$p\left(r\right)=\overset{\min\left(c\;,\;d\right)}{\underset{k=0}{\sum }}\frac{\left(a+b\right)!\left(a+c\right)!\left(c+d\right)!\left(b+d\right)!}{\left(a+k\right)!\left(b-k\right)!\left(c\;-k\right)!\left(d+k\right)!n!}\;$
Output: A list of regulators, each associated with its pseudo activation state and corresponding *p*-value, providing insights into their potential regulatory impact based on the DEG patterns observed within the PKN. 

Once we identified the primary regulatory drivers and significant TFs between the two time points by Algorithms 1 and 2, we used the BFS algorithm to find the shortest paths between all the primary regulatory drivers and TFs. These paths were collected for further analysis.

### 4.6. Temporal Path Searching

In our analysis, we focus on two key aspects of network dynamics: the interconnectivity of nodes and their temporal regulatory shifts. We identify core regulators by selecting nodes that appear in the top 10% by frequency across all examined time points. This criterion ensures that we retain nodes of significant regulatory importance throughout the study period.

We refine our analysis in the temporal dimension by identifying the callback regulation pattern among regulators. Callback regulation is the dynamic shift of a gene from being influenced by upstream signals to becoming a key upstream influencer itself within a defined sequence of time points. The frequent recurrence of such callback events signifies a gene’s pivotal role in regulation, leading to consistent downstream effects upon activation. Our methodology systematically evaluates these events to ascertain the temporal regulatory influence exerted by each gene.

To validate the significance of each callback event statistically, we utilize the Z-test. This method helps us identify genes with significant regulatory influence, beyond just their central position in the network. By applying this approach, our algorithm prunes the network effectively, highlighting pathways critical to *E. coli*’s anaerobic transformation. This process aims to accurately map the network’s structure and dynamics, offering insights into the regulatory mechanisms driving *E. coli*’s metabolic adaptation, as detailed in Algorithm 3.
**Algorithm 3:** Temporal regulation analysis for identifying key regulatory nodes.Procedure: -**Calculate Frequencies:**○**Observed Frequency:** Compute the observed frequency of each node at every time point by counting occurrences in the filtered paths.○**Mean Frequency:** Calculate the mean frequency for each node across all observed paths throughout the dataset. -**Detect Potential Callback Regulators:** For each end node, check if it serves as an upstream regulator within 5 time points. Record such end nodes as potential callback regulators. -**Frequency-Based Node Filtering:** After analyzing all time points, select nodes in the top 10% of frequency. These nodes are considered central to the mechanism network. -**Apply Z-Score Filtering for Callback Regulators:** For each potential callback regulator, perform a Z-test on its observed frequency:○*Null Hypothesis (H_0_):* The frequency of a node does not significantly deviate from the mean frequency, suggesting that any observed changes in node frequency can be attributed to random chance.○*Alternate Hypothesis (H_a_):* The frequency of a node significantly deviates from the mean frequency, specifically after being the target of regulatory action in the preceding time point, indicating a non-random, regulatory effect on the node.Calculate Z-score: (6)$Z=\frac{observed\;frequency-mean\;frequency}{standard\;deviation\;}\;$Determine the *p*-value from the Z-score to assess the statistical significance of deviations. -**Prune the Network:**Maintain nodes in the network that meet one or more of the following conditions:Rank within the top 10% in frequency across all time points.Demonstrate significant upregulation (*p*-value < 0.05) as per Z-score analysis.

### 4.7. Hypergraph Generation

The traditional representations of gene regulatory networks fall short in depicting the true complexity of biological interactions while remaining clear and accessible. The employment of hypergraphs presents a solution that fully encompasses the intricacy of biological systems without sacrificing clarity. This method significantly improves the visualization and analysis of complex gene interactions, providing clearer insights into biological complexities [45].

The community Louvain clustering algorithm [46] is selected for hypernode clustering based on its proficiency in mapping the topological intricacies of networks and conducting modularity optimization, reflecting the interconnected nature of biological systems. Recognizing that gene regulons and protein complexes typically exhibit functional similarities or collective operation, we strategically increase their weighting. This tailored approach ensures that our clustering not only captures the structural essence of the biological network but also underscores the functional coherence among its constituents. Following clustering, we further consulted the GO annotation results to refine hypernode grouping, ensuring compatibility with subsequent functional analyses.

After clustering and validating hypernodes, we transition the results into a PathVisio-compatible format using a custom-developed script [47]. This approach mitigates potential inaccuracies associated with manual network visualization, although manual adjustments to the network’s topological structure remain necessary. These adjustments are essential due to the lack of a standardized mathematical framework capable of automatically guaranteeing an optimal visual representation. This process underscores our commitment to delivering a mechanism network visualization that not only adheres to biological accuracy but also presents the data in a manner that is both accessible and meaningful. 

## Figures and Tables

**Table 1:** The statistical results of PKN construction.

Mechanism Type	Number of Edges from EcoCyc	Number of Edges from RegulonDB	Number of Edges from STRING
Gene–Gene Correlation	0	0	1,089,246
Transcriptional Regulation	8199	4814	0
Protein–Protein Interaction	569	428	582
Metabolic Reactions	5630	0	0

## Data Availability

The original data and code presented in this study are openly available in GitHub at https://github.com/Yigang-Chen/Temporal-causal-modeling (accessed on 8 May 2024). The original experimental data can be accessed through the National Science and Technology Council, Taiwan, under Project No. 102-2815-C-009-025-B.

## Supplementary Materials

The following supporting information can be downloaded at: https://www.mdpi.com/article/10.3390/ijms25115654/s1.
