# Supplementary material for: A Causal Regulation Modeling Algorithm for Temporal Events with Application to Escherichia coli’s Aerobic to Anaerobic Transition
Source: Int J Mol Sci. 2024 May 22;25(11):5654. doi: 10.3390/ijms25115654 (PMC11171773; doi:10.3390/ijms25115654)
Supplement: Supplementary file 1 [file ijms-25-05654-s001.zip › Supplementary Methods.pdf]

**Strains**

E. coli K12 BW25113 (Δ(araD-araB)567, ΔlacZ4787(::rrnB-3), lambda-, rph-1, Δ(rhaD-rhaB)568, hsdR514). BW25113 is a derivative of the F-, λ-, E. coli K-12 strain BD792 CGSC6159. According to the CGSC, BD792 carries rpoS396(Am), but this mutation is not listed in the genotype for BW25113.

**Chemostat growth conditions**

All of the growth condition of this study was modified from previous studies [27]. Detail information about cell culture is described as following. For continuous-culture experiments, a bioreactor (QueueCorp., Parkersburg, W.V.) was fitted with a 10-liter vessel and operated at a 4-liter liquid working volume. A modified Vogel-Bonner medium (pH 6.5) supplemented with Casamino Acids (0.25 mg/liter) and glucose(2.25 mM) was used to limit cell growth. Aerobic continuous culture conditions were maintained by saturating the culture medium with sterile air at a flow rate of 200 ml/min, and anaerobic conditions were maintained by continuously sparging the vessel with oxygen-free nitrogen at a flow rate of 200 ml/min. To vary the degree of air saturation of the medium, the vessel was sparged with a stream of premixed gas in which the proportion of compressed air (21% O2) and compressed nitrogen (99.98%) was controlled by using a manifold with pre calibrated flow meters for each gas. The percent air saturation of the medium was monitored by an Ingold oxygen probe (model1046), which was calibrated with 100% air and 99.98% N2 (0% air) prior to inoculation of the vessel in each experiment. When the chemostat was shifted to a new aeration level, steady state was generally achieved within five reactor residence times. This was confirmed by assaying the b-galactosidase activity of harvested cells as an indicator that gene expression had reached equilibrium. The chemostat was maintained under the same conditions until the β-galactosidase values varied no more than 5%. The b-galactosidase value obtained for each continuous-culture condition was independently determined at least twice, and there was less than 10% variation in b-galactosidase activity. In control experiments performed at 5% air saturation, the flow rate was increased from 200 to 300 ml/min or decreased to 100 ml/min to determine if the gas flow rate affected β-galactosidase levels. Gene expression was unaffected. Thus, the rate of oxygen uptake by the cells was not limiting under these conditions. Another control experiment was performed by increasing. During the experiments, the chemostat was maintained at a medium flow rate(F) of 10 ml/min (cell growth rate, k 5 0.6/h). This corresponded to a cell doubling time (g) of 70 min. The number of cell doublings per hour (m) is equal to 1/g. The cell generation time (g) is equal to (1n 2)/k (15). Between experiments, the chemostat was maintained at a flow rate of 2 ml/min (doubling time, of 5.8 h).

**RNA isolation**

All the samples for transcriptional profiling were harvested at the indicated time points. Chemostat culture samples for transcriptional profiling were directly eluted into RNAprotect (Qiagen) to rapidly stabilize the mRNA. Total RNA was prepared using the RNeasy RNA purification kit (Qiagen), according to the manufacturer's instructions (including the DNase treatment step). RNA was quantified on a NanoDrop.

LB broth (5 ml) was inoculated into the cell culture that was prepared overnight. The overnight culture was diluted 1:100 in LB medium or M9 (with glucose as carbon source at a final

concentration of 2.25 mM) and was incubated at 37 °C until the OD600 of the cell culture reached 0.4 (in the exponential growth phase); 1/10 amount of the RNA stop solution was immediately added into the culture. Cells were harvested by centrifugation at 6000 rpm and 4 °C for 10 min, and the supernatant was removed. Pellets were re-suspended with 1 ml of TRIzol reagent to lyse the cells. The mixture was mixed immediately and incubated in a hot bath at 65 °C for 10 min. To separate the DNA, protein, and RNA, 100 µl of BCP was added. The mixture was vibrated and incubated at room temperature for 15 min and then centrifuged at 12000 rpm and 4 °C for 10 min. The mixture was then separated into three phases, namely, organic phase, interphase, and the colorless aqueous phase. RNA remained exclusive in the aqueous phase, whereas DNA and proteins were in the interphase and organic phase. The 450 µl of aqueous phase was transferred to a fresh tube, and the RNA precipitate from the aqueous phase was added by mixing with 500 µl of isopropanol and 45 µl of 3 M sodium acetate (pH 5.2) into the mixture for precipitation and was stored at −20 °C for at least 2 h. RNA pellet was harvested by centrifugation at 12000 rpm and 4 °C for 30 min and was dissolved with appropriate amount of DEPC-treated water. DNase treatment was conducted by adding 6 µl of 10× DNase I buffer and 6 µl of DNase I at 37 °C for 1 h. Back to the RNA extraction of the precipitation step, the RNA pellet was obtained by centrifugation, and the supernatant was removed. The RNA pellet was washed with 1 ml of 75% ethanol and subsequently centrifuged at 12000 rpm and 4 °C for 10 min, which was repeated twice. Ethanol was removed, and the RNA pellet was dissolved with appropriate amount of DEPC-treated water. RNA quality and concentration were measured by Nanodrop, and A260/280 ratio was about 2.0. RNA samples were stored at −20 °C.

**Reverse transcription**

The first strand cDNA was synthesized by SuperScriptTM II Reverse Transcriptase. About 5 µg of total RNA was mixed with 2 µl of 100 µM random primers, 2 µl of 10 mM dNTP, and DEPC ddH2O to a final volume of 26 µl. The mixture was heated at 65 °C for 10 min and immediately placed on ice for 1 min. About 4 µl of 5× First-Strand Buffer, 2 µl of 0.1 M DTT, 2 µl of DEPC ddH2O, and 2 µl of SuperScriptTM II RT enzyme were added to the total RNA mixture. The mixture was incubated as follows on a PCR machine: 25 °C, 5 min; 50 °C, 60 min; 55 °C, 60 min; 70 °C, 15 min; 60 °C, 30 s; 50 °C, 30 s; 40 °C, 30 s; 25 °C, 30 s; and 4 °C. The cDNA products were stored at −20 °C.

**cDNA preparation and whole genomic array**

The total RNA was extracted using the TRIzol reagent and treated with RNase-free DNase I. The quality of RNA was determined by spectrophotometry. RNA was converted into cDNA incorporating amino-ally dUTP by using SuperScriptTM II Reverse Transcriptase. After removing the RNA template by adding NaOH (1 N) and HCl (1 N), the cDNA was purified by QIAquick Nucleotide Removal kit (QIAGEN) and then converted to Cy5-labeled cDNA. The differential expression of RNA between the WT and topA* mutant strains was measured by single-fluorescence microarray hybridization experiments. Prior to hybridization, the Cy5-labeled cDNA was mixed with hybridization buffer (Phalanx), heated to 95 °C for 5 min, and then maintained at 65 °C. The obtained working solution was infused into pre-warmed assembled chip and hybridized at 42 °C for 16 h. The probed array was then dissembled and

washed with buffer I (2× SSC, 0.2% SDS), followed by buffer II (2× SSC) at hybridization temperature for 5 min, and buffer II at room temperature for another 5 min. The probed chips were rinsed with buffer III (0.2× SSC) and then dried by centrifugation. Microarray CEL files were background corrected, normalized, and expression value-calculated using the Robust Multi-chip Average algorithm, resulting in log2 expression values.

**Quantitative real time PCR mRNA expression level**

Real-time PCR was used for gene expression analysis. The experiment was performed in a 96-well plate (Roshe, LightCycler® 480 Multiwell Plate 96, white). Each 20 µl reaction volume contained 10 µl of 2× SYBR Green (Roshe, LightCycler® 480 SYBR Green I Master), 2 µl of 0.1× cDNA, 6 µl of nuclease-free water, and 2 µl of 10 mM pre-mixed primers (forward and reverse primers). Real-time PCR was conducted by RosheLightCycler® 480 System, and the data were analyzed by LightCycler 480 software version 1.5.0.39 and 2nd derivative Max. 16s rRNA and nanK were used as internal control and positive control, respectively. Experiments were conducted in triplicate.
