# Supplementary figures and images for: A Causal Regulation Modeling Algorithm for Temporal Events with Application to Escherichia coli’s Aerobic to Anaerobic Transition
Source: Int J Mol Sci. 2024 May 22;25(11):5654. doi: 10.3390/ijms25115654 (PMC11171773; doi:10.3390/ijms25115654)

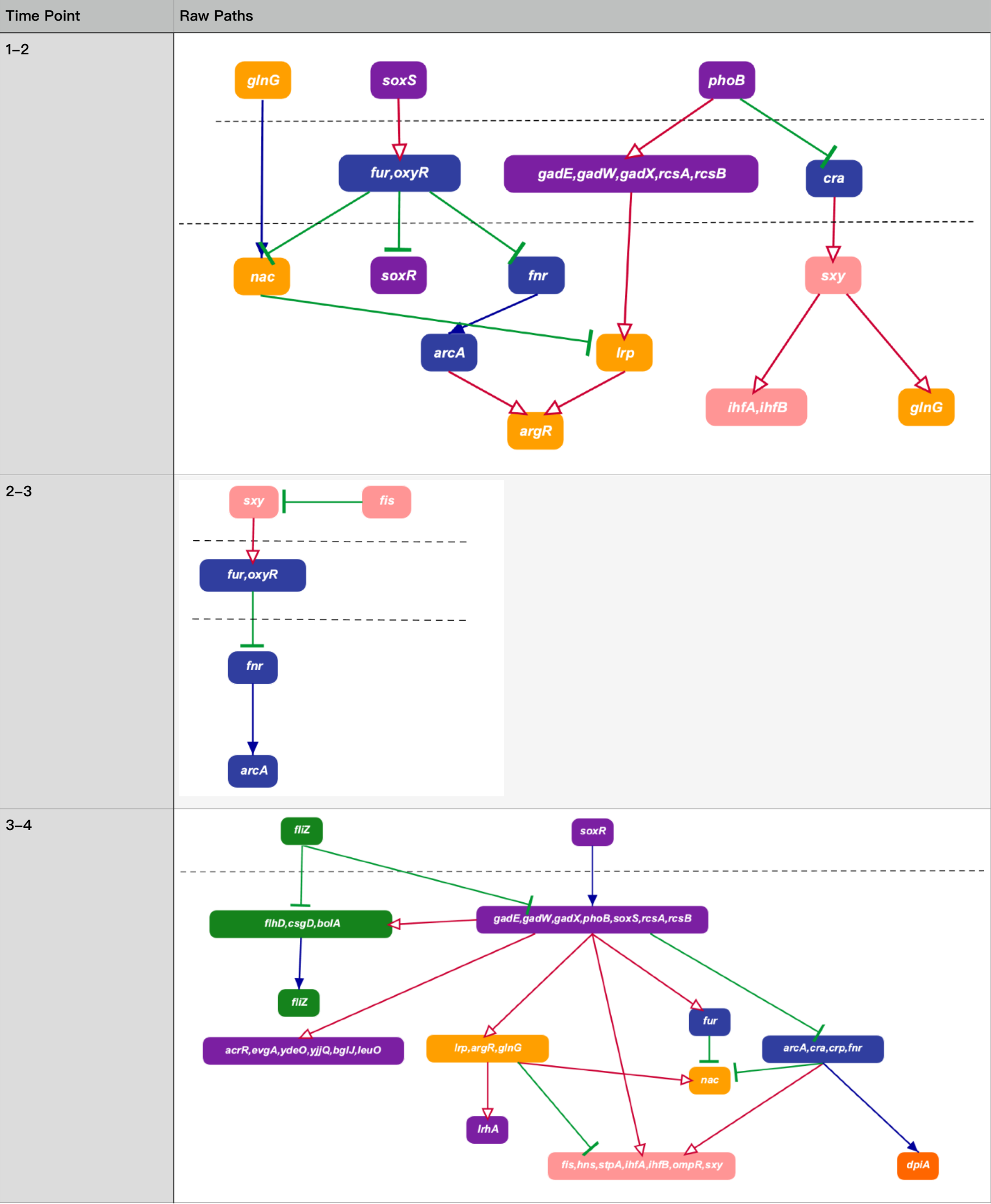

Time Point

Raw Paths

4-5

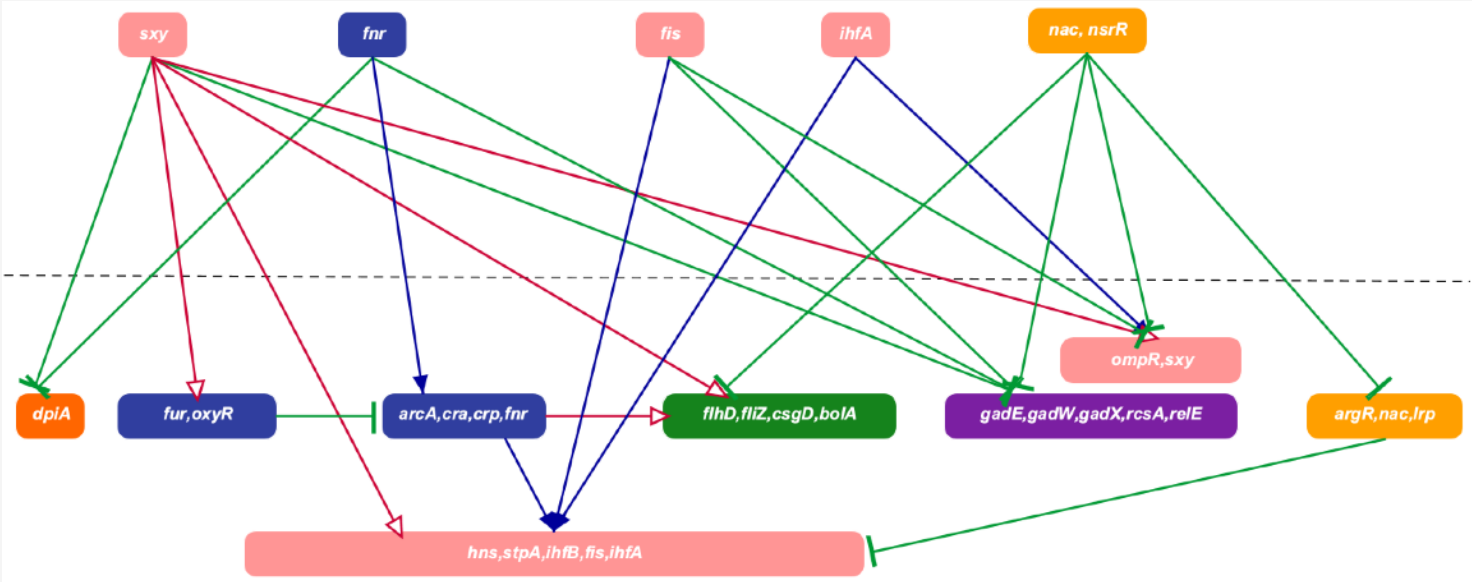

5-6

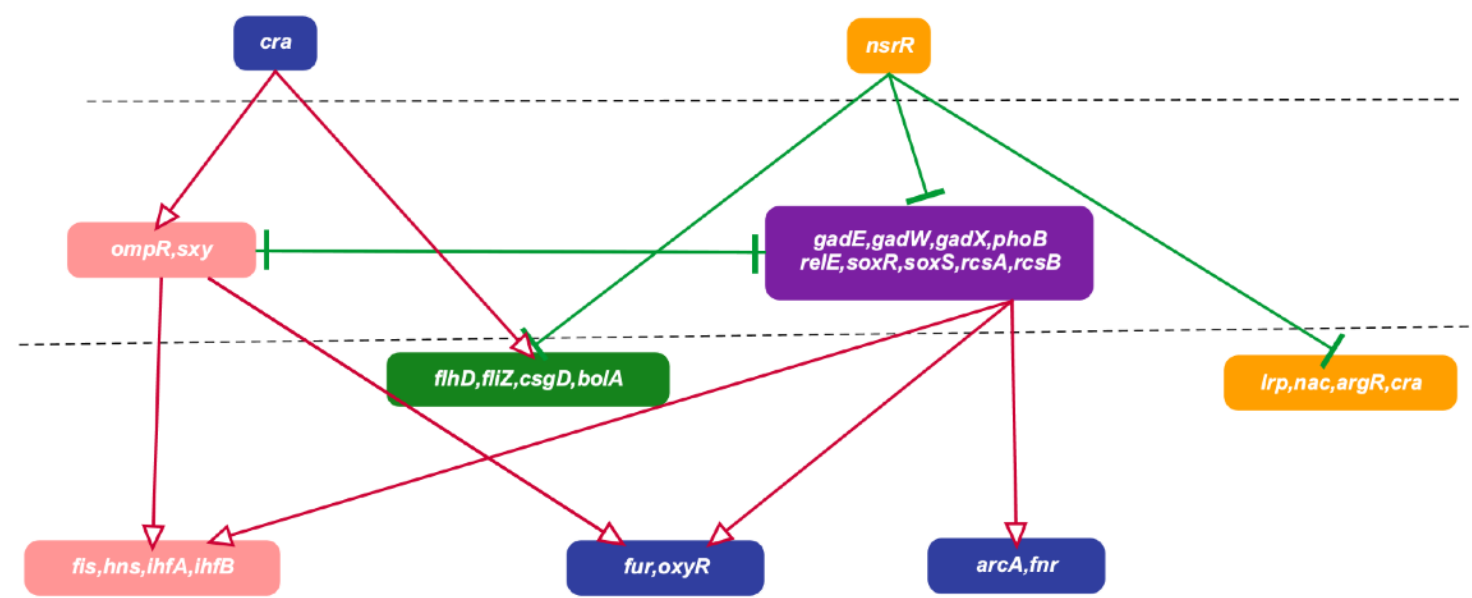

6-7

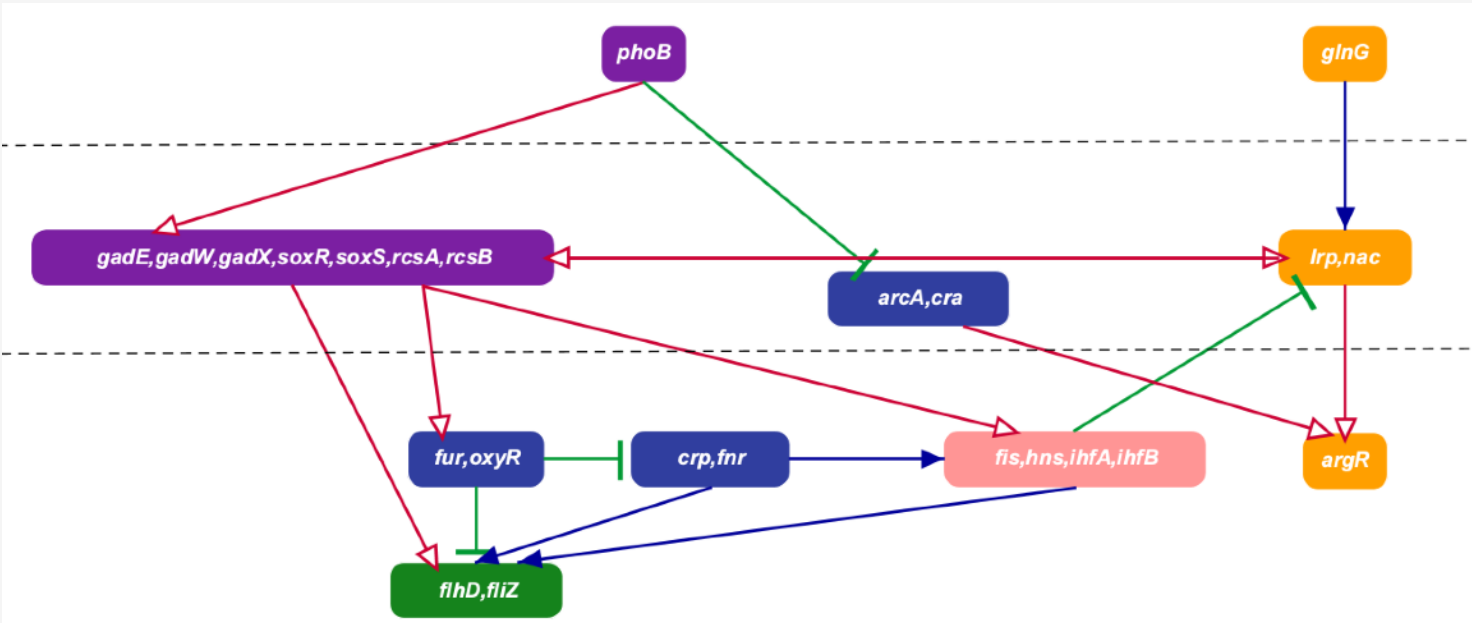

7-8

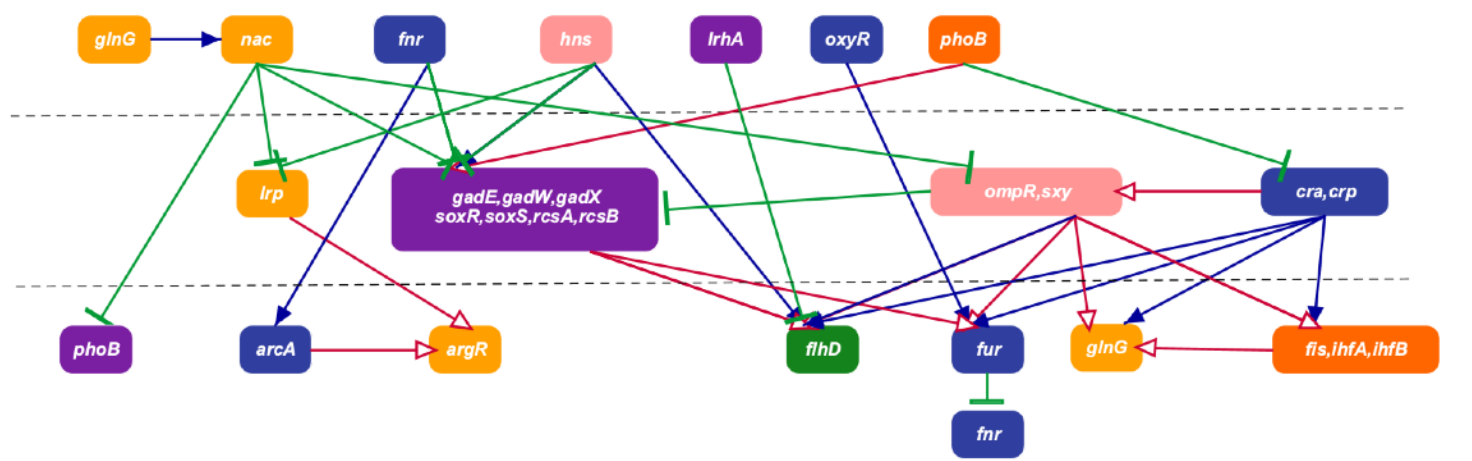

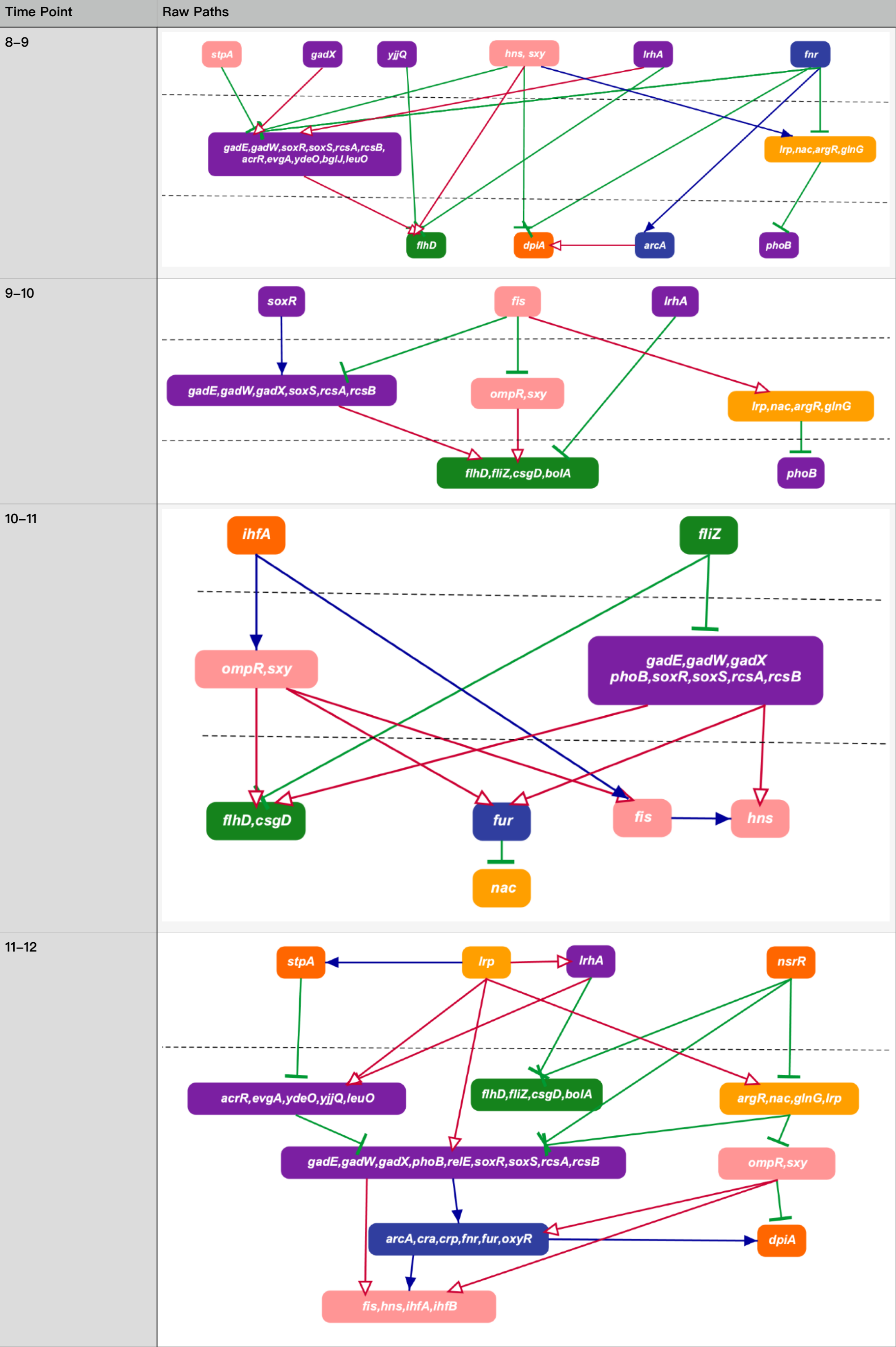

| Time Point | Raw Paths |
|------------|-----------|
| 12-13      |           |
| 13-14      |           |
| 14-15      |           |

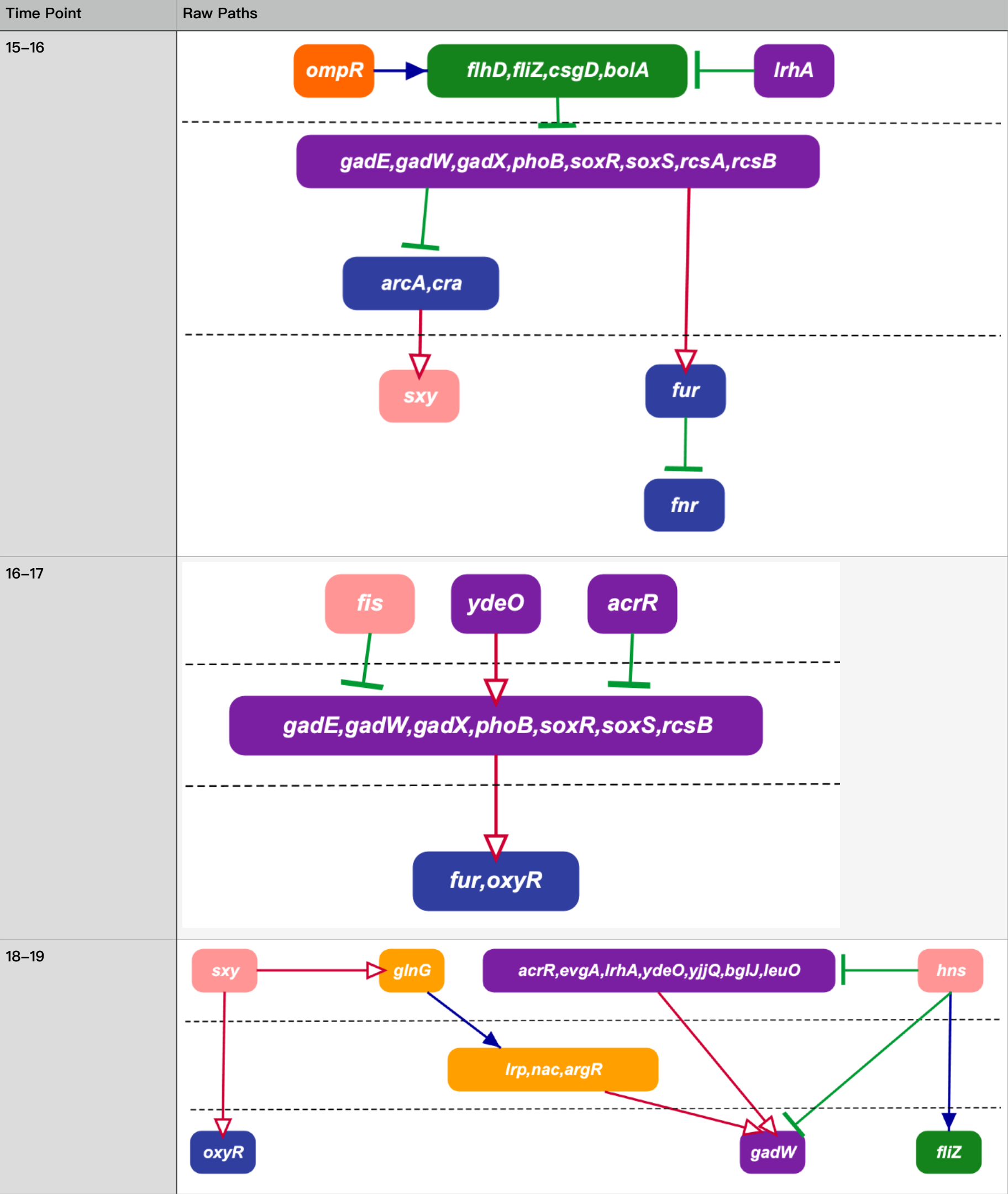

Supplement: Supplementary file 1 [file ijms-25-05654-s001.zip › Supplementary File.pdf]
